# Effect of a Blend of *Zingiber officinale* Roscoe and *Bixa orellana* L. Herbal Supplement on the Recovery of Delayed-Onset Muscle Soreness Induced by Unaccustomed Eccentric Resistance Training: A Randomized, Triple-Blind, Placebo-Controlled Trial

**DOI:** 10.3389/fphys.2020.00826

**Published:** 2020-07-21

**Authors:** Diego Dominguez-Balmaseda, Ignacio Diez-Vega, Mar Larrosa, Alejandro F. San Juan, Nicolas Issaly, Diego Moreno-Pérez, Silvia Burgos, Manuel Sillero-Quintana, Cristina Gonzalez, Andrea Bas, Marc Roller, Margarita Pérez-Ruiz

**Affiliations:** ^1^Research Group on Nutrition, Physical Activity and Health, Faculty of Biomedical Sciences Universidad Europea, Madrid, Spain; ^2^Research Group on Exercise, Health and Applied Biomarkers, Faculty of Sport Sciences, Universidad Europea de Madrid, Madrid, Spain; ^3^Faculty of Physical Activity and Sport Sciences-INEF, Universidad Polit cnica de Madrid, Madrid, Spain; ^4^Nicolas Issaly, Valencia, Spain; ^5^Departamento de Educación, Métodos de Investigación y Evaluación, Universidad Pontificia de Comillas, Instituto Católico de Artes e Industrias-Instituto Católico de Administración y Dirección de Empresas, Madrid, Spain; ^6^Natural Origins, Lozanne, France

**Keywords:** annatto, ginger, young athletes, pain, sport performance, recovery, ReWin(d), heart rate variability

## Abstract

**Background:**

There is an increasing interest in the use of eccentric muscle exercise to improve physical condition, especially with regards to its health-related benefits. However, it is known that unaccustomed eccentric exercise causes muscle damage and delayed pain, commonly defined as “delayed onset muscle soreness” (DOMS). The efficacy of herbal preparations in subjects suffering from DOMS has been reported in a few previous studies with small or moderate outcome measures related to muscle recovery. The present study aimed to evaluate the effects of a polyherbal mixture containing whole *Zingiber officinale* Roscoe and *Bixa orellana* L., powders called ReWin(d), in young male athletes suffering from DOMS induced by a 1 h session of plyometric exercises.

**Methods:**

Thirty-three young male athletes participated in this randomized, Triple-blind, placebo-controlled trial: 17 of them assigned to the ReWin(d) group and 16 of them to the placebo group. Creatine kinase (CK) was measured as a muscle damage marker, pain was assessed using the Visual Analog Scale (VAS), muscle performance was measured through half-squat exercise (HS) monitored with an accelerometer (Encoder), and heart rate variability (HRV) was monitored for 5 min with the subjects in the supine position. All determinations were performed before and after the eccentric session and 24, 48, and 72 h after the session.

**Results:**

The eccentric exercise session caused an increase in CK at 24 and 48 h after exercise intervention in both groups (*p* < 0.001). There was no interaction between groups regarding muscle damage. The pain increased after the training session in both groups (*p* < 0.001), and a significant interaction was observed between groups at 48 h after exercise (*p* = 0.004). Lower limb muscular power showed a significant interaction between groups 24 h after exercise (*p* = 0.049); the placebo group showed a reduction in muscle power compared to the ReWin(d) group. The LF/HF ratio decreased significantly at 72 h after exercise in the herbal group but not in the placebo group.

**Conclusion:**

The herbal supplement maintained the maximum power of the lower limbs and attenuated muscle pain.

**Clinical Trial Registration:**

www.ClinicalTrials.gov, identifier NCT03961022.

## Introduction

Eccentric resistance training is widely used by coaches due to its beneficial effects on the athlete’s performance. Eccentric action can be integrated into different types of muscle training. Plyometric exercises, such as drop jump, are frequently used to improve speed and jumping ability in athletes. One of the most important benefits of this kind of training is the rapid muscle strength and muscle mass gain, with rapid neuronal adaptations that increase athlete’s performance (Hody et al., 2019). As a result, this type of exercise with a predominance of an eccentric contraction is very useful for the prevention and treatment of different chronic diseases, in which the maintaining of the muscle mass is essential for a good clinical course of the disease. This type of contraction provokes a molecular activation signaling of the muscle satellite cells and the stimulation of the anabolic signals that generate muscle hypertrophy (Hyldahl and Hubal, 2014; Hyldahl et al., 2014; Douglas et al., 2017). Evidence suggests that eccentric resistance training is superior, in terms of muscular hypertrophy, over concentric or conventional strength training (Julian et al., 2018). However, unaccustomed eccentric resistance training causes mechanical damage, which triggers the loss of calcium homeostasis, with possible inflammatory reaction and reactive oxygen species (ROS), as well as delayed onset of muscle soreness (DOMS) that is characterized by mechanical hyperalgesia, rigidity, swelling of the exercised area, and decreased muscle function. DOMS are usually felt during palpation, contraction, or stretching of the affected muscle. It normally appears between 12 and 24 h after an unusual eccentric resistance training, with a maximum peak from 24 to 72 h. Naturally, DOMS progressively decreases, and it disappears within 5–7 days after exercise (Nosaka and Newton, 2002).

The analysis of the recovery process up to 72 h after the eccentric resistance training helps coaches to know when athletes could receive a new safety and effective high-intensity stimulus and not to hinder muscle adaptation mechanisms. This recovery time can be studied by analyzing the exercise performance and also the sympathetic-parasympathetic influx, measured through the heart rate variability (HRV). The sympathetic activity of the central nervous system increases during exercise and decreases during the recovery period; therefore, previously suppressed parasympathetic activity becomes dominant during recovery and reduces HRV (Kingsley and Figueroa, 2016).

The need to perform eccentric resistance training to achieve positive adaptations is inherent to the development of DOMS, but a solid and consistent treatment for DOMS has not been established to date. Although there are multiple practices for DOMS alleviation, only a few of them have scientific support (Connolly et al., 2003; Heiss et al., 2019). The most well-known therapeutic programs for the management of DOMS include cryotherapy, stretching, massage, use of compression during or after exercise, ultrasound muscular treatment, or oral non-steroidal anti-inflammatory drugs (NSAIDs) (Matsumura et al., 2015). Nowadays, the use of NSAIDS to attenuate DOMS is not advisable, as they have gastrointestinal, renal, and hepatotoxic side effects, and their effectiveness to treat DOMS is not clearly demonstrated (Feucht and Patel, 2010).

The use of dietary supplements by athletes has increased in the last decade (Sellami et al., 2018). Several ergogenic dietary supplements [for example, taurine, branched-chain amino acid (BCAA) powder, vitamins, polyphenols, n-3 polyunsaturated fatty acids (PUFAs), fish oil, chondroitin sulfate] may help to improve muscle function and reduce symptoms of DOMS (Harty et al., 2019) and to enhance vascular homeostasis and DNA repair mechanisms in elite triathletes (García-Flores et al., 2016, 2018). In sport, emerging evidence suggests that the chronic consumption of ginger may act to mitigate postexercise soreness (Wilson, 2015). Due to its antioxidant and anti-inflammatory properties, the use of ginseng, curcumin, green tea, *Rhodiola rosea*, and ginger extract have been suggested to reduce markers of muscle damage (Harty et al., 2019). The use of raw ginger supplementation to alleviate arm muscle pain induced by eccentric exercise has shown moderate to large reductions in muscle pain (Black et al., 2010). The anti-inflammatory activity of [6]-gingerol (6-GN), one of the main pungent compounds in fresh ginger, has been attributed to the inhibition of proinflammatory cytokines and nitric oxide and COX-2 enzymes (Tripathi et al., 2007; Wilson, 2015). In the same vein, bixin and norbixin, the main carotenoids present in *Bixa orellana* (annatto), have been attributed with biological antioxidants, anti-inflammatory, and analgesic properties (Shahid-ul-Islam Rather and Mohammad, 2016). Given the antioxidant, analgesic, and anti-inflammatory properties of both herbal products, a new natural herb blend that associates *Zingiber officinale* Roscoe rhizome powder with *B. orellana* L. seed powder, was designed to study its effect on DOMS. The specific objectives of this study were to evaluate the effect of the herbal supplement on local pain, performance, and recovery after an eccentric exercise session in healthy young athletes. We hypothesized that, compared to placebo, the herbal supplement would accelerate the recovery rate of DOMS, by focusing on muscle function and pain perception and improving the autonomic nervous system through heart rate variability.

## Materials and Methods

### Herbal and Placebo Supplements

A natural organic blend 1:0.75 (*w*/*w*) of, respectively, *Z. officinale* Roscoe rhizome powder (ginger) and *B. orellana* L. seed powder (annatto) called ReWin(d) was supplied by Natural Origins (69380 Lozanne, France). ReWin(d) and placebo pills were indistinguishably packaged, had the same size, content (333 mg) and color, and were labeled with A or B code. The phytochemical analysis of ginger and annatto powders were respectively outsourced to independent laboratories, respectively, the Laboratoire Provençal de Plantes Aromatiques et Médicinales (Avenue de la Gare BP 47 26170 Buis les Baronnies, France) and Eurofins Analytics France (Rue Pierre Adolphe Bobierre BP 42301 F-44323 Nantes Cedex 3, France). The occurrence of gingerols and shogaols content in the ginger powder, including 6-gingerdiol, 6-gingerol, 8-gingerol, 10-gingerol, 6-shogaol, 8-shogaol, and 10-shogaol, was quantified by high-performance liquid chromatography (HPLC), leading to a 2.4% (*w*/*w*) total content of gingerols and shogaols expressed as capsaicin. The content of bixin and norbixin in the annatto powder was quantified by high-performance liquid chromatography (HPLC) leading to 1.32% (*w*/*w*) content of bixin and norbixin. Placebo pills were composed of wheat maltodextrin (Natural Origins, Lozanne, France).

### Subjects

Participant’s recruitment was carried out among students of the Faculties of Physical Activity and Sport Sciences and Physiotherapy of the Universidad Europea de Madrid. Participants were included in the study when they met the following criteria: (i) being male and practicing physical activity three times a week for at least the last year [assessed by the International Physical Activity Questionnaire (IPAQ) questionnaire), (ii) being between 18 and 35 years old, (iii) not taking statins or cyclosporine, (iv) no diagnostic pathology or chronic disease, and (v) not having musculoskeletal injury in lower limbs in the last 6 months, and (vi) not being smoker.

### Study Design

The study protocol adhered to the principles of the Declaration of Helsinki (“World Medical Association Declaration of Helsinki: ethical principles for medical research involving human subjects”), and it was approved by the Regional Committee of Ethics of Research with Medicines (CEIM) of the Community of Madrid (reference number: 47/734814.9/18). All participants were informed about the procedures of the interventional trial, and they agreed to sign a written informed consent form before entering the study. The report of the results has been elaborated following the Consolidated Standards of Reporting Trials (CONSORT) Statement for Reporting Randomized, Controlled Trials of Herbal Interventions (Gagnier et al., 2006).

**FIGURE 1:**
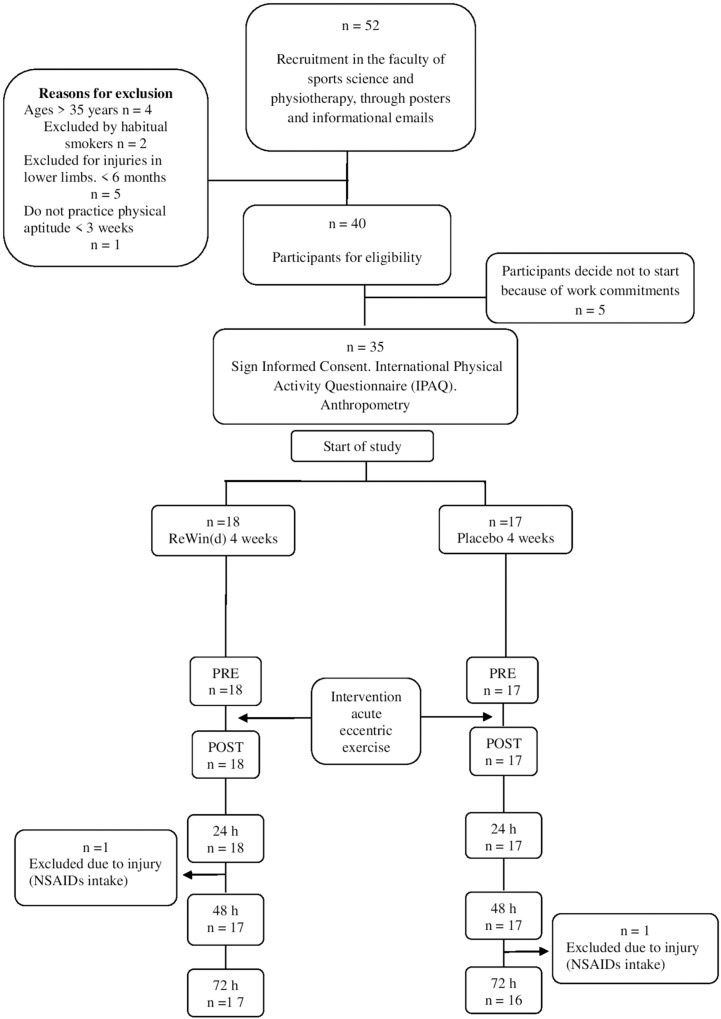
Study flow chart.

A randomized, triple-blind, placebo-controlled trial involving 40 healthy and physically active men aged between 18 and 35 years was conducted. The triple blind allowed neither the participants nor the principal investigator nor the data analyst to know the allocation to each of the situations. From the 52 people contacted, 40 met the inclusion and exclusion criteria, and 35 of them were finally enrolled in the study (Figure 1). To calculate the sample size, a previously pilot study was conducted with a mixed design of three repeated measures (pre, post, and 24 h) and two groups, the variable used was the effect of the power of the lower limb measured with the half squat exercise. Assuming an α error = 0.05, a β error = 0.2, and an effect size η^2^*_*p*_* = 0.013, it was determined that a sample of *n* = 36 was needed (G-Power v.3.1 software; Faul et al., 2009). Anticipating a loss of 10% of follow-up, 40 participants were enrolled in the study. Randomization of the groups was carried out with the random function of Microsoft Office Excel (Microsoft Corporation, Redmond, Washington, United States); participants were assigned to herbal supplement group [ReWin(d)] or placebo group (Placebo). Once participants were enrolled in the study, demography data (age) and anthropometric (weight and height) data were recorded. The weight (kg) was measured with a scale (Ano Sayol SL, Barcelona, Spain) and the height (cm) with a stadiometer (Asimed T2, Barcelona, Spain). The body mass index (BMI) was calculated as weight (kg)/height^2^ (m).

**FIGURE 2:**
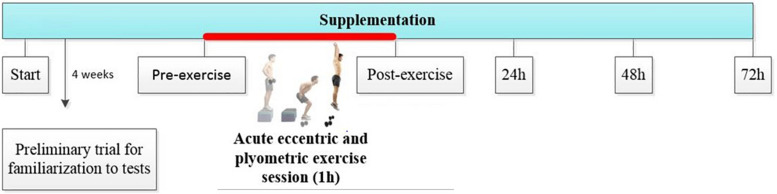
Study design and intervention scheme. Pre–Post: times before and after 1 h acute eccentric and plyometric exercise intervention. Post24, Post48, and Post72: evolution and behavior of variables during the 24, 48, and 72 h of recovery.

Participants began to take supplement or placebo pills, according to the random assignment, 4 weeks before the exercise intervention and the next 3 days after the acute exercise session. Subjects took six capsules a day (two with breakfast, two with lunch, and two with dinner). Every week, subjects were contacted by email and/or by phone to record any possible adverse effect. During the 4 weeks of supplementation, participants became familiar with evaluation tests. Recruitment, degree of adherence, randomization, and loss of subjects during the study are summarized in Figure 1. After 4 weeks, basal levels for all studied variables were taken before the intervention (Pre), immediately after the exercise session (Post), at 24 h (Post24), at 48 h (Post48), and 72 h (Post72). A scheme of the study design and intervention is showed in Figure 2.

### Eccentric Exercise-Induced Muscle Damage Protocol

Participants conducted a 60 min acute session, performing plyometric and eccentric exercises to cause DOMS according to the study of Howatson et al. (2012). The acute exercise session consisted of 10 sets of 10 vertical countermovement jump repetitions from a 50 cm step, holding dumbbells with 10% of the subject’s body weight. Participants had 15 s of rest between repetitions and 60 s of rest between sets. After that, they performed a circuit of 3 eccentric exercises and 3 sets of 10 repetitions; first exercise was “Nordic hamstring,” second was to run 10 s in open kinetic chain suspension (subject was held with his hands on a bar and extended arms), and finally, the “backward kick” at the same time the trunk advances while remaining parallel to the ground and the subject holds 10 kg of weight with the arms.

### Creatine Kinase Activity

The creatine kinase (CK) measurements were performed in whole venous blood by venepuncture in the antecubital vein using EDTA-vacutainer^®^ tubes (BD, NJ, United States). The blood sample was placed on the reactive CK bands (Reflotron CK, Roche, Germany) with a pipette (Applicator 9609800, HIRSCHMANN Laborgeräte, Eberstadt, Germany) (μg/L) and then into the automatic reflexion photometer (Reflotron Plus, Roche, Germany) (± 0.2% accuracy and an uncertainty of ± 0.5% derived to the utilized instruments) and the CK strips of the same commercial brand. The person did not need to fast; however, it was important to avoid any type of strenuous physical exercise at least 2 days before conducting the test to avoid any interference in CK levels (Baird et al., 2012).

### Muscle Performance

The lower limb muscle performance was evaluated through maximum power efforts with the half squat exercise (HS). Before the tests, the participants performed a 5 min warm-up on a cycle ergometer, followed by ballistic stretching of the lower limbs and five repetitions of HS at 50% of the subject’s body weight. The procedure was supervised by an expert researcher. Then, the maximum mechanical power developed at a given load (80% of the bodyweight) was evaluated in HS exercise. Each subject performed two repetitions with 3 min of rest. The load that corresponds to the 80% of the bodyweight was set on the first laboratory session (Pre), and then, the same load was repeated in Post, Post24, Post48, and Post72 times. The subjects stand with feet placed at a position that matched the width of the hip and the beam in the rear side of the deltoid with hands clutching the barbell, and then, they flex the knees to 90° followed by knees extension to the original standing position (Bogdanis et al., 2014). The test was performed at maximal speed in a multipower, bar-guiding system Smith machine using 1. 25-, 2. 5-, 5-, 10-, and 20-kg disks (Technogym, Gambettola, Italy). In this setup, both ends of the barbell were fixed allowing only vertical movement of the bar. To estimate the execution velocity of each repetition, a triaxial accelerometer (Sensorize C1-P, Free Power, Sensorize, Italy) was used. The accelerometer was attached to one end of the bar to avoid hindering the HS movement. This system allows for the measurement of the vertical displacement of the bar according to the exercise movement. Data were analyzed using the system’s software Freepowernext (Amer Sports, Helsinki, Finland, and the bar velocities (average and peak) in m/s, and the powers (average and peak) in watts were obtained. The analysis of both variables at different times can detect power losses and increased fatigue as determinants of muscle performance.

### Pain Assessment

The Visual Analog Scale (VAS) is a valid approach to measure the intensity of pain that the subjects describe, with the maximum reproducibility among observers (Hawker et al., 2011). The VAS consisted of a horizontal line of 10 cm, at whose ends were the extreme scores of the pain symptoms: pain “0” will be the absence of pain and “10” an unbearable pain (Haefeli and Elfering, 2006). The participants indicated at the end of the HS test at each time point (Pre, Post, Post24, Post48, and Post72) their perception of the pain.

### Heart Rate Variability Analysis

Heart rate variability (HRV) is the result of the interactions between the autonomous nervous system and the cardiovascular system. HRV analysis was performed with a heart rate monitor (Ambit3, Suunto, Amer Sports, Strava, Finland), for 5 min with the subject in the supine decubitus position on a stretcher in a quiet and soft light environment (Flatt and Esco, 2016). The subjects were asked not to speak or move. Cardiac electrical signals were monitored with a band located in the thorax. The temperature in the laboratory was maintained between 22 and 24°C. HRV represents variations between consecutive heartbeats (beat to beat or R-R interval) over time. The disappearance of variations between consecutive heartbeats is a result of autonomic dysfunction. Data were analyzed with the Kubios HRV Stands 3.1.0 software for Windows (Biomedical Signal and Medical Imaging Analysis Group, Department of Applied Physics, University of Kuopio, Finland), and three parameters were determined: standard deviation of all NN intervals in millisecond (SDNN), low frequency (LF), high frequency (HF), and the index for the sympathovagal balance as the ratio of the low to high-frequency power (LF/HF).

### Statistical Analysis

Statistical testing was carried out using SPSS v.21 (IBM, Armonk, NY). Shapiro–Wilks test was used to determine the normal distribution of the data. Homoskedasticity and sphericity were checked. When the assumptions were met, a mixed ANOVA 5 × 2 was carried out, and the significance level was set at *p* = 0.05, adjusting multiple comparisons with the Bonferroni test. Otherwise, non-parametric Friedman or *U* Mann-Whitney tests were used, adjusting α error with Bonferroni correction (*p* = 0.007). Means with standard deviations were used for parametric analysis, and median with first–third quartiles were used to non-parametric analyses. The effect size was estimated with the Cohen’s *d* parameter, being *d* < 0.20 trivial effect, *d* < 0.50 small effect, *d* < 0.80 medium effect, and *d* ≥ 0.80 large effect.

## Results

### Baseline Data

The demographic characteristics of the participants did not differ between individuals of both groups (Table 1).

**TABLE 1:** Characteristics of the study participants.

	**Total**	**ReWin(d) (*n* = 17)**	**Placebo (*n* = 16)**	***p***
Age (year)	22.93.9	223.3	23.94.3	0.195
Height (m)	1.790.07	1.790.07	1.780.08	0.369
Weight (kg)	76.4111.27	76.2511.05	76.5911.83	0.782
BMI (kg/m^2^)	23.92.6	23.72.8	24.12.4	0.546
Energy expenditure (MET/week)	4,8802,820	4,5972,716	5,1812,979	0.503
Sedentarism (min/week)	364137	340144	390127	0.463
Training experience (year)	9.97.7	12.28.3	7.46.2	0.067
Training days/week	4.71.1	4.71.1	4.81.1	0.987
Training hours/session	1.20.4	1.40.5	1.10.2	0.096

**Results are expressed as mean ± standard deviation.**

To control that the response to the eccentric exercise session was homogeneous for both groups, the CK activity was monitored (Table 2). An increased in CK was observed 24 and 48 h after exercise intervention in both groups (*p* < 0.001), and no interaction between the groups was detected respect to pre (Post, *p* = 0.173; Post24h, *p* = 0.463; Post48h, *p* = 0.375; Post72h, *p* = 0.657), post (Post24h, *p* = 0.708; Post48h, *p* = 0.838; Post72h, *p* = 0.533), post 24 h (Post48h, *p* = 0.973; Post72h, *p* = 0.657), or post 48 h (Post72h, *p* = 0.127).

**TABLE 2:** Creatine kinase whole blood levels at different time points.

	**Pre**	**Post**	**Post24**	**Post48**	**Post72**
ReWin(d) (IU/L)	94.95 (83.3–211)	131.5 (90.5–288)	335 (174–587)*	233 (124–380)*	168 (98.1–290)
Placebo (IU/L)	66.5 (56.8–94.3)	99.9 (67.8–121)	171 (144–341)*	164 (102–201)*	110.25 (68.25–215.5)

Results are expressed as the median (quartile 1–3). *Significant differences with respect to pretime.

### Pain

**FIGURE 3:**
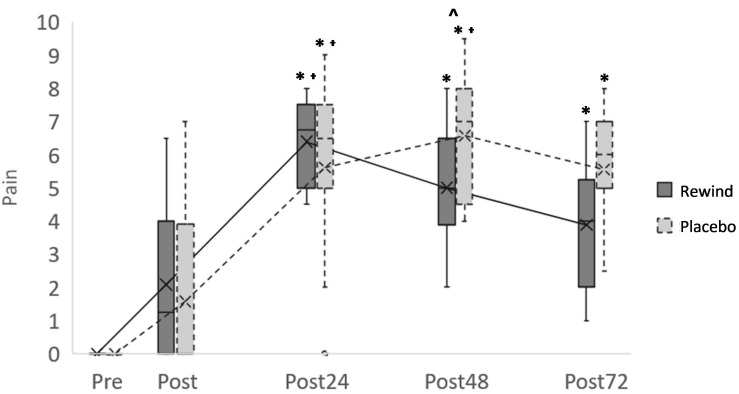
Pain evolution after exercise comparing ReWin(d) and Placebo groups. Significant pain differences (*p* < 0.007) with respect to T1(^∗^), with respect to T2(ɫ), and between ReWin(d) and Placebo (^∧^).

Pain perception increased after the eccentric exercise session in ReWin(d) group (*p* < 0.001) and in Placebo group (*p* < 0.001) (Figure 3). Both groups felt more pain at Post24, Post48, and Post72 times after exercise in comparison to pre-exercise (Pre) and also in comparison to Post24h, indicating an increment of pain immediately after exercise that was maintained at least until the next 3 days. When the effect of the supplements was analyzed, it was observed that the Placebo group presented significant pain at Post24 in comparison to Post, whereas ReWin(d) group did not. Thus, significant differences between ReWin(d) and Placebo were observed at 48 h postexercise (*p* = 0.004; *d* = 0.82); although differences at 72 h postexercise were still moderate, attending to effect size, they were not significant (*p* = 0.011; *d* = 0.50). No differences between groups were found at Pre, Post, or Post24 times.

### Muscle Performance

**FIGURE 4:**
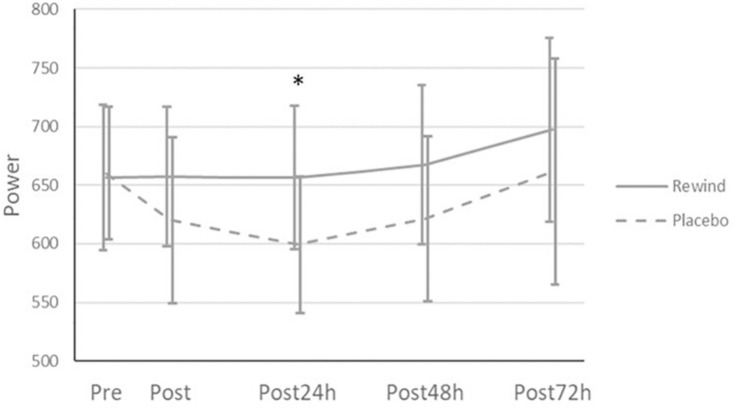
Lower limbs power evolution after exercise comparing ReWin(d) and Placebo groups. *Significant differences (*p* < 0.05).

Lower limbs power performance in HS (Figure 4) showed significant interaction between ReWin(d) and Placebo groups in the first 24 h (*p* = 0.049; *d* = 0.63). The Placebo group moderately reduced its muscle power at 24 h (*p* < 0.05) compared to the ReWin(d) group. However, significant interaction disappeared in the subsequent times (*p* = 0.482; *d* = 0.33), due to the natural process of muscle recovery.

### Heart Rate Variability

No significant differences were found in the SDNN for any of the groups (Table 3). The LF/HF ratio decreased significantly between post and 72 h after exercise in ReWin(d) group (*p* = 0.004) but not in the Placebo group (*p* = 0.249). No significant interactions were found (*p* > 0.007) between groups, although moderate to large effect was found between pre-post (*d* = 0.75) and post-24 h (*d* = 0.88) in LF/HF ratio (Table 3).

**TABLE 3:** Results of heart rate variability throughout the study.

		**Pre**	**Post**	**Post24h**	**Post48h**	**Post72h**	***p* intra**
SDNN	ReWin(d)	64.1 (49.7–79.3)	39.3 (23.7–58)	63.5 (45.2–69.2)	50.8 (42.5–69.8)	69.4 (50.7–110.3)	0.007
	Placebo	53 (44.7–68.1)	37.3 (29.1–51.8)	60.7 (54.4–65.3)	58.2 (38.5–76.7)	52.6 (45.7–80.5)	0.055
	*p*.inter	0.281	0.736	0.852	0.709	0.25	
LF/HF	ReWin(d)	1.5 (0.7–3.1)	3 (2.1–6.2)	1.7 (1.1–2.1)	1.5 (1.3–3.9)	1.3 (1–2.1)ɫ	0.004
	Placebo	1.9 (1.6–2.7)	2 (1.4–3.4)	2.1 (1.9–3.3)	1.6 (0.9–2.1)	1.3 (0.9–2.4)	0.249
	*p* inter	0.347	0.058	0.04	0.518	0.861	

*ɫDifferences with respect to Post (p < 0.007). SDNN (ms), the standard deviation of the interbeat interval of normal sinus beats. LF/HF ratio of lower frequency to high frequency power.*

### Adverse Events

No adverse events were detected throughout the study.

## Discussion

The results obtained, in this randomized controlled triple-blind intervention trial, demonstrate that the ReWin(d) supplement attenuates pain originated after unaccustomed eccentric resistance exercises in trained youths. The ReWin(d) group also achieved an early recovery over time compared to the placebo group. This would indicate that the muscular responses that follow this type of acute exercise that originate from DOMS are not hindering, as those subjects supplemented with ReWin(d) maintain a better level of exercise performance at 24 h compared to the placebo group.

Different hypotheses have been raised to explain the development of DOMS after a trained session of strength exercises with the participation of eccentric contraction. Among them, the increase in inflammation and oxidative stress that accompany the practice of exercise is the most noticeable (Peake et al., 2007; Suzuki, 2018). The proposed ReWin(d) supplement is the blend of two natural plants (ginger and annatto) with demonstrated anti-inflammatory, antioxidant, and analgesic actions (Black et al., 2010; Wilson et al., 2015; Shahid-ul-Islam Rather and Mohammad, 2016; Roehrs et al., 2017; Wilson, 2018). The decrease in pain caused by supplementation with ReWin(d) may be due to the inhibitory effect of ginger on cyclooxygenase-2 and therefore on the production of prostaglandins and other inflammatory mediators such as certain cytokines (Kim et al., 2017). In fact, the 12-week intake of 1.5 g/day of ginger significantly decreased the levels of different inflammatory cytokines [tumor necrosis factor (TNF)-alpha, interleukin (IL)-6, and IL-1beta] after a treadmill test (Zehsaz et al., 2014). Another mechanism through which ginger could decrease pain is because some of its components, such as shogaols, which are agonists of the type 1 transient potential cation channel receptors (TRPV1) of the central and peripheral nervous system involved in the transmission and modulation of pain (Iwasaki et al., 2006; Hitomi et al., 2019). However, there is no conclusive evidence for the antioxidant effect of ginger (Ranchordas et al., 2017). For this reason, annatto, which has demonstrated antioxidant effects (Shahid-ul-Islam Rather and Mohammad, 2016; Roehrs et al., 2017;

Conte et al., 2019), was selected to be part of the supplement. The excessive inhibition of the oxidative stress signal produced by the eccentric exercise can lead to inhibiting exercise adaptations, diminishing consequently the beneficial effects associated with the practice of exercise (Gross and Baum, 2015). For this reason, the ideal supplement is the one that has in its composition antioxidant and anti-inflammatory components capable of attenuating the occurrence of DOMS and not inhibiting the adaptations associated with exercise. According to the results obtained in this work, we can state that ReWin(d) supplement has beneficial effects on the muscle recovery of athletes subjected to eccentric work without impairing the response caused by exercise. Furthermore, no associated adverse effects have been found during the present study, which is another advantage, as many athletes use NSAIDs to attenuate DOMS despite the associated side effects of this type of drugs (Feucht and Patel, 2010; Chabbey and Martin, 2019). The advantage of the herbal supplement ReWin(d) is that it is a raw vegetable supplement composed of powders of whole plants. Whole vegetable powder provides synergy and balance of nutrients and active compounds to work together for the health benefit (Williamson, 2001). To the best of our knowledge, the present study is the first one that uses the combination of both compounds in the form of a plant and not an extract and that analyzes the effect of the supplement for 72 h after performing an acute exercise with a large component of eccentric contraction capable of causing DOMS.

Exercise with a predominance of eccentric contraction causes greater improvements in muscle strength, power, and speed, compared to concentric or traditional training (Douglas et al., 2017).

In the present study, the power was measured through HS, observing that after an acute session of eccentric exercise, the exercise performance at 24 h decreased in the Placebo group and was maintained in the ReWin(d) group, indicating that the ReWin(d) supplement can prevent the loss of short-term performance. Our results are in line with previous findings in which ginger prevented delayed muscle damage in the flexor of the elbow of men and women without weight training (Matsumura et al., 2015). The faster recovery in the ReWin(d) group could be related to a faster recovery of the vagal sympathetic system in the ReWin(d) group, as it is suggested by the moderate to large effect size found. The faster recovery of the vagal sympathetic system would allow an earlier recovery of the internal homeostasis environment in the ReWin(d) group as can be observed in the analysis of the HRV. The training session can be considered as stress for the body that causes alterations in the internal homeostasis and modulation of the autonomic nervous system. The changes in the activity of the autonomic nervous system are manifested by increased sympathetic and/or decreased parasympathetic activity, which is reflected by heart rate variability parameters (Kingsley and Figueroa, 2016). The analysis of HRV in this study indicates a moderate effect size on the recovery of sympathetic-vagal balance within 24 h of acute exercise in the group supplemented with ReWin(d) compared to the placebo group. The use of HRV as a biomarker of response to different components or types of diet has been recently proposed (Young and Benton, 2018). Different studies have shown an effect of omega 3 fatty acids on parasympathetic activity (Xin et al., 2013), of the polyphenols of red wine in the SDNN (Janszky et al., 2005), and of the Ginsenoside Rb1, an active compound of *Panax ginseng*, on the increase in the LF/HF ratio (Chang et al., 2015). However, the effects on HRV of ginger, annatto, or a mixture of both have not been previously approached. To our knowledge, this is the first time in the literature that the effect of ginger and annatto supplementation on the recovery of the sympathetic-vagal balance is described. Recently, HRV has reached a growing interest in sports science, mainly to control training loads and as an instrument to learn about the recovery process of the autonomic nervous system after exercise. A better knowledge of the recovery process would allow trainers to more efficiently plan the exercise load and also to anticipate subsequent training sessions. At the moment, it is known that the athletes should avoid high-intensity exercise in the 72-h postexercise since an insufficient recovery period can contribute to overtraining and more easily cause a muscle injury (Cheung et al., 2003; Cipryan et al., 2017; Dupuy et al., 2018), but probably with the use of this type of supplements as ReWin(d) together with the HRV analysis, trainers could anticipate their exercise sessions. This study has some limitations; predictably, the high variability presented by the participants in the maximal power of the lower limbs prevented confirming the differences between groups at the postexercise, post-48 h, and post-72 h times. Another limitation was that we have used CPK as an indirect marker of muscle damage; although this is the most commonly used marker in routine analysis, it is not the most sensitive marker for detecting muscle damage This marker also presented high variability that prevented demonstrating differences in response to exercise between groups. In addition, the study has not assessed markers related to inflammation and antioxidant effect of the supplement; although these were not the objectives of our work, future work should be considered in this regard.

## Conclusion

The present study demonstrated that supplementation with ReWin(d) supplement (2 g/day) in trained young men helps to maintain the maximum power of the lower limbs and attenuate the pain muscle caused by unaccustomed eccentric exercise. These improvements could be associated with the autonomic nervous system response, although this finding must be confirmed in future research.

## Data Availability Statement

The raw data supporting the conclusions of this article will be made available by the authors, without undue reservation, to any qualified researcher. Requests to access the datasets should be directed to Margarita Pérez-Ruiz, margarita.perez@universidadeuropea.es.

## Ethics Statement

The study protocol adhered to the principles of the Declaration of Helsinki (World Medical Association, 2002) and was approved by the Regional Committee of Ethics of Research with Medicines (CEIM) of the Community of Madrid (reference number: 47/734814.9/18). The patients/participants provided their written informed consent to participate in this study.

## Author Contributions

MP-R, ML, NI, DM-P, and MR conceived and designed the study. DD-B, AS, MS-Q, ID-V, AB, and SB acquisition and analyzed the data. ID-V, MP-R, ML, NI, DD-B, and CG interpreted the data. MP-R, ML, DD-B, NI, and CG drafted the manuscript. ML, MR, and DD-B revised the final manuscript. All authors contributed to the article and approved the submitted version.

## Conflict of Interest

MR and NI were employed by the company Natural Origins. The remaining authors declare that the research was conducted in the absence of any commercial or financial relationships that could be construed as a potential conflict of interest.
